# Formulation of precise exercise intervention strategy for adolescent depression

**DOI:** 10.1002/pchj.726

**Published:** 2024-02-01

**Authors:** Xianghe Chen, Xinyu Zeng, Chi Liu, Pengcheng Lu, Ziming Shen, Rongbin Yin

**Affiliations:** ^1^ College of Physical Education Yangzhou University Yangzhou China; ^2^ Physical Education and Sports School of Soochow University Suzhou China

**Keywords:** depression, mental health, precise exercise, teenagers

## Abstract

The high incidence of adolescent depression has become the focus of social and academic attention. Exercise is an important method to improve adolescent depression, but its intervention effect is still controversial. This study first compares and analyzes the relevant studies at home and abroad and finds that exercise prescription in adolescent depression intervention is not accurate enough. A meta‐analysis was conducted to develop a precise exercise intervention strategy for adolescent depression. Firstly, this thesis identified how to optimize five elements (exercise intensity, exercise frequency, exercise time, exercise cycle, and exercise type) of exercise prescription to improve depression in adolescents. This is the problem. Furthermore, the concept of “precision exercise” was proposed, and a precision exercise intervention strategy (moderate‐intensity aerobic exercise for 8–10 weeks, 3 times/week, 45–50 min/time) was constructed to improve adolescent depression. This paper also presents research that strengthens the cross‐sectional research and empirical research on adolescent depression and establishes a precision exercise prescription database for adolescent depression in China. In conclusion, this study not only puts forward the concept of “precision exercise” but also constructs a precision exercise intervention strategy for adolescent depression, which has important theoretical and practical significance for improving the high incidence of adolescent depression.

## INTRODUCTION

The coronavirus pandemic has led to a high incidence of mental health problems, such as depression among teenagers (Al‐Halbusi et al., 2022; Rahmat et al., 2022). Research has found the incidence of depression among adolescents in China was 15.4%, with a total number of more than 30 million. The incidence of suicidal thoughts (suicidal ideas) among middle school students was 17.7% (Huang et al., 2019). The high prevalence, high mortality, and serious harmfulness of adolescent depression have brought great negative effects on themselves, their families, and society. Therefore, the *Plan of the* “*Healthy China 2030*” clearly put forward that we should strengthen the intervention in common mental disorders and psychological and behavioral problems such as depression to promote the comprehensive development of adolescents' physical and mental health. Based on the above, adolescent depression has been listed as a major social issue of national concern, as well as a focus of scientific research.

Exercise is an important method to prevent and treat adolescent depression, and aerobic exercise, resistance exercise, and group exercise are the most common methods. In 1984, McCann and Holmes (1984) confirmed the beneficial effect of aerobic exercise on anti‐depression. Moreover, aerobic exercise exhibited better anti‐depression performance than traditional medical intervention, but the dose–response and effect of aerobic exercise on depressed adolescents remains to be clarified. It has been reported that aerobic interval training with an 80% maximum heart rate (MHR) in 10 days could significantly improve adolescent depression (Knubben et al., 2007). However, Blumenthal et al. (2007) found that the effect of short‐term exercise on adolescent depression was not obvious, while the effect of aerobic exercise (70%–85% MHR, 30 min/time, 3 times/week, and over 4 months) on adolescent depression was similar to that of antidepressants. In another study, medium‐intensity aerobic exercise (dynamic cycling) for 3–4 days/week and at least 9 weeks could effectively reduce depression. However, when exercise duration was less than 4 weeks, less than or equal to 1 time per week, and with low or high intensity, the improvement in depression would significantly reduce (Meyer et al., 2016; Schmitter et al., 2020). The results indicated that the antidepressant effect of moderate and high‐intensity aerobic exercise for a relatively long duration was significantly better than that of low‐intensity aerobic exercise. Moreover, the precise combination of exercise type, exercise frequency, and exercise intensity had a more significant effect on improving depressive behaviors (Thabrew et al., 2018). Compared with competitive basketball, non‐competitive basketball (12 weeks, 45 points/time, 1 time/day, 2 days/week) significantly improved reduced the depressive symptoms in junior high school students (Yang, 2012). Regression analysis indicated that male college students with severe depression experienced more peer exclusion than female counterparts, and participating in individual sports would aggravate depressive symptoms, while group sports had a significant improvement in symptoms (Perron‐Gélinas et al., 2017). In addition, the occurrence of depressive psychological problems and exercise behavior of adolescents are also affected by the level of health education, and individuals' perception of their own psychological problems and the benefits of exercise regulation encourage them to cope through exercise (Azadi et al., 2021). The COVID‐19 pandemic, social media overuse, and other factors have caused the high incidence of depression in adolescents, making the improvement effect of exercise more important (Abbas, 2020; Abbas et al., 2019; Zhou et al., 2022).

The above analysis found that the role of exercise in improving depression in adolescents is more controversial. This is closely related to the inaccurate formulation of exercise prescriptions for adolescent depression; that is, how to delicately formulate the five elements of exercise prescription (exercise intensity, exercise frequency, exercise time, exercise cycle, and exercise type) has become an urgent scientific problem to be solved. Based on this, this study aimed to conduct research into the following three aspects: sorting out the research on exercise intervention for adolescent depression; proposing the concept of “precision movement” for the first time; and conducting a meta‐analysis of the effects of exercise on depression in adolescents. Furthermore, the aim is to construct a precise exercise prescription strategy for the best exercise type, exercise intensity, exercise frequency, exercise time, and exercise cycle for adolescent depression. The results of this study can provide an effective theoretical and methodological basis for the prevention and intervention of adolescent depression.

## OVERVIEW OF EXERCISE INTERVENTION RESEARCH ON ADOLESCENT DEPRESSION

### History of exercise intervention for adolescent depression

By the 1980s, the study of exercise and depression had been attracting more and more attention from researchers. Because of the fundamental correlation between the two studies, they soon intersected and extended a new research direction: exercise improves depression. Martinsen et al. (1985) found that 9 week aerobic exercise could increase the maximum oxygen uptake of elderly patients with mental disorders and had an antidepressant effect at the same time. Although this study was relatively rough, it confirmed the potential benefits of exercise in improving depression, which laid the foundation for the majority of studies on exercise and adolescent depression. From this early stage, the concept of “treating” adolescent depression by exercise gradually sprang up. After 2000, the related research in this field gradually increased, focusing on the role of exercise in improving the drug dependence of depressed adolescents. It was found that 12‐week aerobic exercise with medium intensity could effectively improve the symptoms and drug dependence. Petty et al. (2009) published a study on the effects of self‐regulation intervention on adolescent mental health in *JAMA Psychiatry* and they found that physical exercise could improve the symptoms of adolescent depression. This study, which adopted a longitudinal study instead of the more common cross‐sectional study used in the previous studies, made the conclusion that physical exercise could improve adolescent depression more convincing, and also received great attention from the academic community. Another landmark achievement was a meta‐analysis published in 2010, which analyzed all the published studies (22 items) on exercise improving adolescent depression, and it was found that the effect of exercise on adolescent depression could reach a medium effect size (Fabricatore et al., 2011). This study had a great impact, and its research method has been referenced by most scholars in this field. From that stage, the concept of exercise improving adolescent depression has been rapidly developed and spread, and the quantity and quality of research have been significantly increased. In the past decade, researchers have tried to explore the effect of exercise on adolescent depression from two aspects: the specific type of exercise and the different type and intensity of exercise types on adolescent depression. In the aspect of the effect of different exercise intensity on adolescent depression, some scholars found that some exercise effects were not significant or even had no impact when exploring different exercises to improve adolescent depression (Li et al., 2019; Matsuzaki et al., 2016). Since exercise exhibited significant heterogeneity in improving adolescent depression, “precise exercise” for adolescent depression should be urgently studied.

### History of exercise intervention for adolescent depression in China

Xiao Yunqi first proposed that running was a “good medicine” to prevent and treat depression in 1988 in China (Song, 1990). The research on exercise improving adolescent depression underwent three stages: in the first stage (1988–2004), researchers tried to use exercise to intervene in depression and explored its effect (Zhang, 1989). This stage laid a theoretical foundation for exploring how exercise could improve adolescent depression. During this period, a few studies were reported in China, and it was proposed that the antidepressant effect of physical exercise depended on exercise intensity, duration, and frequency (Xiang, 1992). In the second stage (2005–2011), on the basis of an in‐depth exploration of the mechanism and theory of depression, the study of physical exercise improving the psychological effect of adolescents clearly indicated that the medium‐intensity group projects that lasted for more than 10 weeks, 2–5 times a week, 20–40 min/time, had the most obvious beneficial performance on improving depression (Yang, 2012). In the third stage (2012–present), the “*Healthy China 2030*” *Planning Outline* issued in 2016 clearly proposed to strengthen the intervention of common mental disorders and psychological and behavioral problems such as depression so as to promote the comprehensive development of adolescents' physical and mental health. In 2017, 22 departments, including the National Health and Family Planning Commission, issued the first macro‐guidance document in the field of mental health, the *Guiding Opinions on Strengthening Mental Health Services*, which clearly pointed out that “exercise intervention is the priority method to improve adolescent depression”, and raised the improvement of adolescent depression and other mental diseases by exercise to the national strategy.

In addition, the outbreak of COVID‐19 has caused a high incidence of depression in adolescents, which makes the improvement effect of exercise even more important (Aqeel et al., 2022; Shoib et al., 2022). At this stage, the number and quality of studies on exercise improving adolescent depression increased prominently. For example, some scholars explored the impact of non‐competitive basketball on the depression of middle‐school students, and they found that non‐competitive basketball was a convenient, feasible, and effective way to alleviate and eliminate adolescent depression (Yang, 2012). Moreover, researchers employed resting functional magnetic resonance imaging (fMRI) technology to study and analyze the correlation between abnormal functional activity (neurocognitive network function) in the posterior brain area and suicide attempts in adolescent depression patients and the intervention effect of physical exercise (Pan et al., 2017). Chinese scholars explored the improvement effect of different physical exercise methods on adolescent depression patients with different severity disease levels for the first time (Wang C., 2014). After that, some scholars were concerned that physical exercise could improve the depression‐like behaviors caused by related diseases (such as sleep disorders) (Sun, 2019). Unfortunately, these studies only focused on the exercise types, which led to the result that in the process of improving the depression symptoms through physical exercise, teenagers with depression often had to deal with the lack of relevant physical exercise types, the poor effect of improving depression, and the long time spent on physical exercise. Under the current background of the high prevelence of adolescent depression in China, it is increasingly necessary to explore depression and its corresponding best exercise method from a wider perspective in order to build a targeted, efficient, and low‐cost exercise intervention model to improve adolescent depression in China, that is, the construction of a precise exercise improving adolescent depression model.

### Existing issues

Although the effect of exercise on improving adolescent depression symptoms has been confirmed, the following questions remain to be answered: (1) What exercise intensity has the best intervention effect? (2) Which exercise frequency has the best intervention effect? (3) Which exercise duration per section has the best intervention effect? (4) Which the duration of exercise program has the best intervention effect? (5) Which exercise type has the best intervention effect? Therefore, the concept of precision exercise and precision exercise intervention strategies for adolescent depression should be urgently studied. Based on the above, our study aims to put forward the concept of “precise exercise” for the first time and to employ meta‐analysis to analyze the current research on the improvement of adolescent depression by exercise, explore the precision exercise intensity, exercise frequency, time per section, intervention duration, and exercise type to improve adolescent depression. It is hoped that this study can provide precise exercise prescription strategies for the improvement of adolescent depression.

## META‐ANALYSIS ON PRECISE EXERCISE INTERVENTION STRATEGY FOR ADOLESCENT DEPRESSION

### Proposing the concept of precise exercise

Precise exercise refers to a personalized, refined, and targeted exercise formulation model based on adolescent depression + individualized exercise and on the characteristics of adolescent depression such as exercise habits and physical functions. Unlike the previous “one‐size‐fits‐all” exercise intervention, formulating precise exercise prescriptions would make fine adjustments and variations to the prescribed exercise prescriptions for different patients under this model.

### Construction of precise exercise intervention strategy for adolescent depression

#### Data source and retrieval method

Seven databases were searched, including MEDLINE, PubMed, Web of Science, The Cochrane Library, China National Knowledge Infrastructure (CNKI), Wanfang Database, and Weipu Database. The search period was from the establishment of the database to 30 May 2022.

**FIGURE 1:**
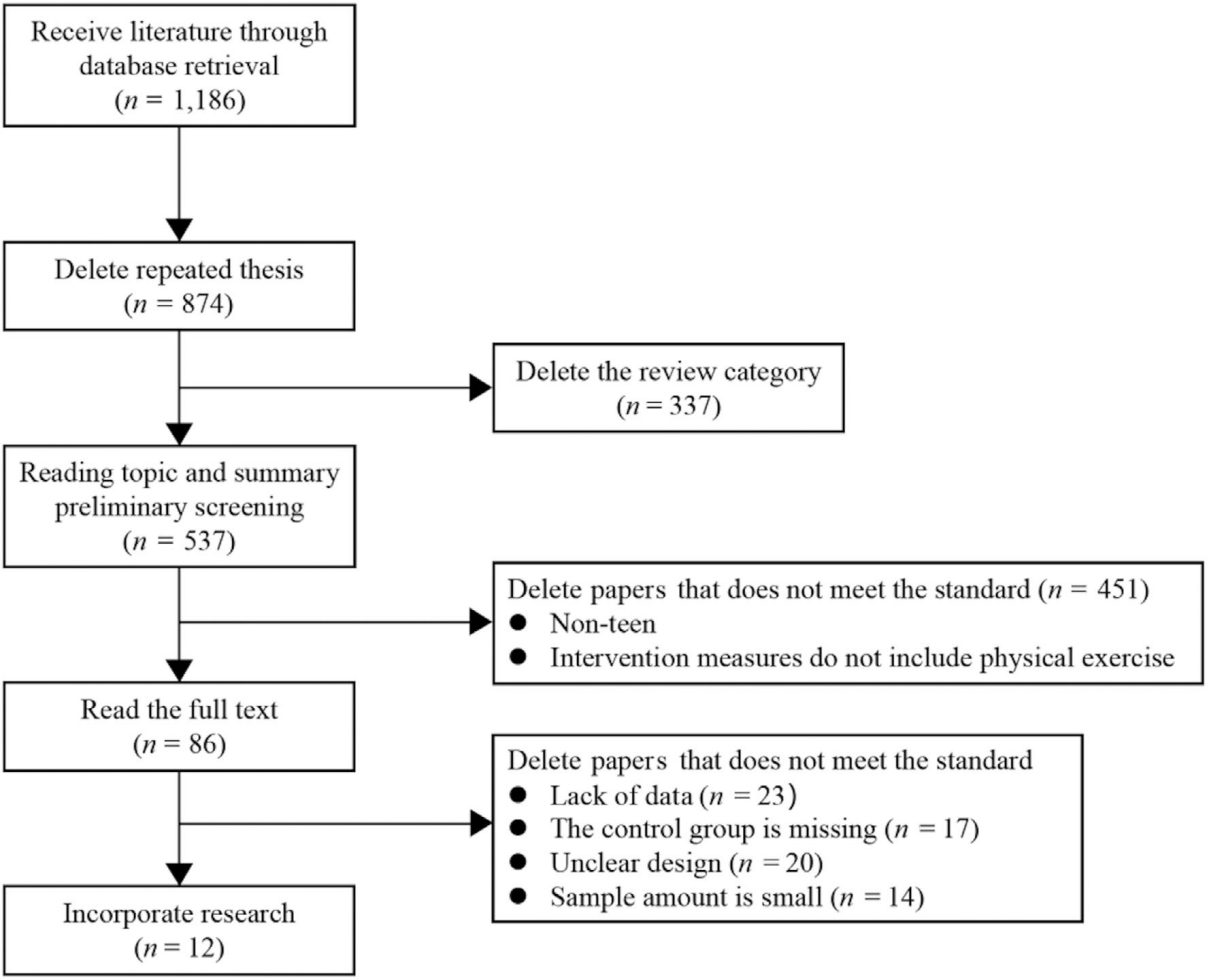
Schematic diagram of document retrieval and screening process.

The retrieval strategy involved a combination of subject and free words and was finalized after repeated checks. Chinese search terms included exercise intervention, physical exercise, physical activity, depression, adolescents, and middle‐school students. English search terms included depression, exercise intervention, physical exercise, sports intervention, and adolescent. The retrieval process is shown in Figure 1.

#### Inclusion and exclusion criteria of the study

##### Inclusion criteria

Based on the PICOS (Population, Intervention, Comparison, Outcome, Study design) algorithm used in evidence‐based medicine (Higgins et al., 2008), the inclusion criteria were as follows: (1) Population: adolescents aged 12–19 years. (2) Intervention: compared with the control group, the experimental group received a structured exercise program (exercise type, exercise duration, exercise intensity, exercise frequency, or exercise period). (3) Comparison: the control group did not receive exercise intervention. (4) Outcome: specific study data (such as mean, standard deviation, 95% confidence interval, etc.). (5) Study design: randomized controlled trials (RCTs); original full texts of peer‐reviewed manuscripts in Chinese or English, available by 30 May 2022.

##### Exclusion criteria

Exclusion criteria were as follows: (1) Population: any identified physical or non‐depressive psychiatric illness, aged over 12–19 years old. (2) Intervention measures: combined intervention measures, such as exercise combined with nutrition supplements or music therapy. (3) Comparison: lack of control group. (4) Outcome: data could not be extracted, or raw data could not be obtained by contacting the corresponding author. (5) Study design: There was a lack of description of physical exercise (exercise intensity, exercise frequency, exercise time, exercise cycle, or exercise type) in the intervention design.

#### Data extraction and document quality evaluation

##### Literature screening

Two authors then independently screened the literature according to the inclusion and exclusion criteria. Firstly, articles were preliminarily screened by reading titles and abstracts, and those that did not meet the inclusion and exclusion criteria were removed and recorded. In the second step, the full text of the remaining articles was downloaded, read, and reviewed, and the articles were re‐screened. In case of disagreement between the two authors, a third author reviewed and decided whether to include the study.

##### Data extraction

Two authors extracted and recorded the following data using a predesigned data extraction form: (1) Basic article information: name of the first author, study country, and year of publication. (2) Participant information: depression measures (depression or depressive symptoms), age, sex ratio, sample size. (3) Physical exercise variables (such as exercise intensity, exercise frequency, exercise time, exercise cycle, or exercise type).

##### Quality assessment

The risk of bias (quality) of randomized controlled trials (RCTs) was assessed using the assessment tool in Review Manager 5.4 developed by Cochrane. In case of disagreement, a third author assessed the issue and discussed it until consensus was reached. This tool evaluates the risk of bias in six aspects: selection bias, performance bias, measurement bias, follow‐up bias, reporting bias, and other bias, and finally evaluates the study quality as low risk of bias, high risk of bias, and unknown risk of bias.

### Statistical analysis

Review Manager 5.4 software was employed to analyze the outcome indicators. Due to the differences in the measurement tools used for depression indicators, the standardized mean difference (SMD) and 95% confidence interval (CI) were used as the main effect measures in this study. *Q* statistics and *I* ^2^ statistics measured the heterogeneity of the included study. When the heterogeneity test result was *p* > .1 or *I* ^2^ < 50%, it indicated that the included study had no heterogeneity, and the fixed effect model was used for analysis. When the heterogeneity test result was *p* < .1 or *I* ^2^ > 50%, it could be considered that the included study was heterogeneous, and the random effect model was used for analysis.

### Results

#### Basic characteristics of the included study

The COVID‐19 pandemic (Global Burden of Disease 2019 Under‐5 Mortality Collaborators, 2021), differences in environmental quality (Iorember et al., 2022), and lifestyle changes (Mohammadi et al., 2021) all lead to a high incidence of mental health problems such as depression in adolescents. In order to explore the role of exercise in improving adolescent depression, this study first analyzed the relevant papers included in this study, as shown in Table 2; according to Table 1, 12 studies were included in this study – four Chinese and eight English –all of which were controlled experiments with pretest and posttest design. There were 462 adolescents with depression, aged between 12 and 18 years. Self‐rating depression scale (SDS), Beck depression inventory (BDI), children's depression inventory (CDI), and other scales were employed to evaluate depression symptoms. The type of exercise intervention was divided into group exercise and individual exercise. The exercise duration was 30–75 min, the duration of intervention was 4–14 weeks, and the exercise frequency was 2–5 times a week.

**TABLE 1:** Basic characteristics of the included study.

References	Country	Age (year)	Sample size, *N* (female %)	Exercise group				Sample content (E/C)	Outcome measures
				Type	Exercise program duration (week)	Exercise session duration (min)	Frequency (sessions/week)		
Carter et al., 2016	United Kingdom	14–17	21 (81)	Individual sports	6	60	2	44/43	CDI‐2
Hughes et al., 2013	United States	12–18	11 (42)	Team sports	12	60	3	16/14	CDRS‐R
Philippot et al., 2022	Belgium	14–17	25 (63)	Team sports	6	50	4–5	20/20	HADS‐D
Daley et al., 2006	United Kingdom	11–16	28 (56)	Team sports	14	30–40	3	26/24	CDI
Khalsa et al., 2012	United States	15–19	51 (42)	Team sports	11	30–40	3	14/12	BASC‐2
Mohammadi, 2011	Iran	NR	NR	Individual sports/Team sports	8	75	3	80/40	BDI
Dabidy‐Roshan et al., 2011	Iran	15–18	152 (100)	Individual sports	6	NR	3	12/12	HAMD
Wunram et al., 2018	Germany	12–18	46 (72)	Individual sports/Team sports	6	30	3–5	35/17	DIKJ
Wang & Liu 2018	China	14–16	95 (51)	Team sports	12	45	4	90/98	PHQ‐9
Liang et al., 2021	China	12–17	22 (55)	Team sports	4	15	2–3	20/20	SDS
Han et al., 2020	China	12–17	43 (51)	Team sports	8	45	4	42/42	HAMD
Wang, 2017	China	14–18	16 (67)	Team sports	10	40	5	12/12	SCL‐90

Abbreviations: BASC‐2, behavior assessment system for children, 2nd edition; BDI, Beck depression inventory; C, control group; CDI‐2, children's depression inventory 2; CDRS‐R, children's depression rating scale‐revised; E, exercise group; HADS‐D, depression subscale of the hospital anxiety and depression scale; HAMD, Hamilton depression scale; NR, not report; PHQ‐9, patient health questionnaire 9; SCL‐90, symptom checklist 90; SDS, self‐rating depression scale.

#### Literature quality evaluation

**FIGURE 2:**
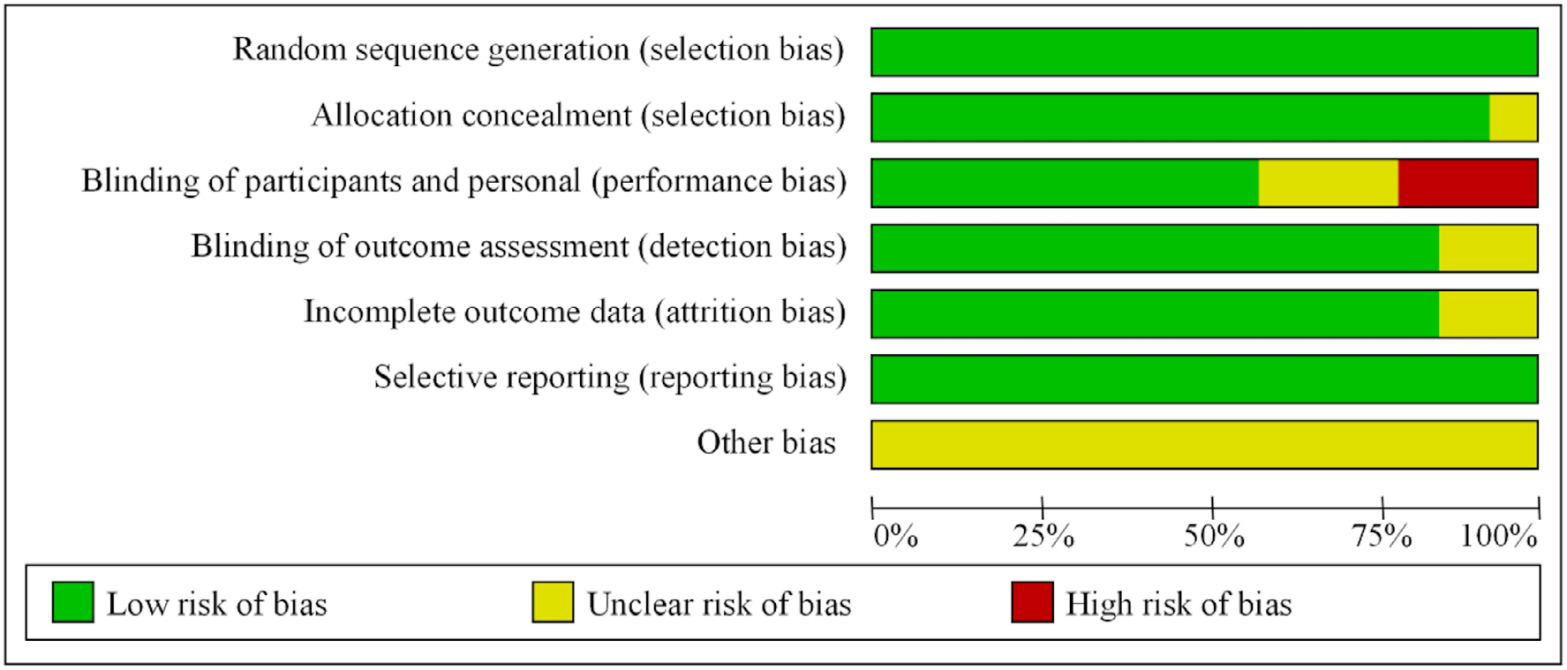
Schematic diagram of bias risk ratio.

**FIGURE 3:**
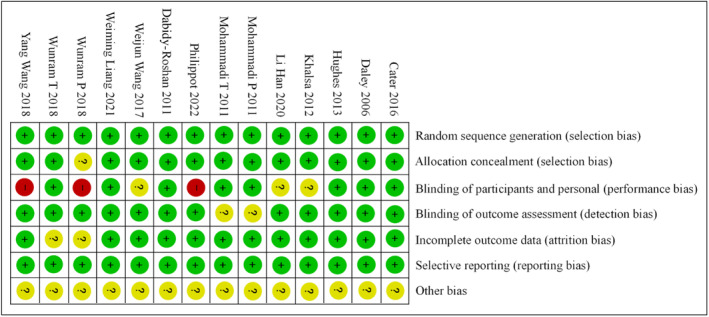
Schematic diagram of bias risk assessment.

In order to ensure the accuracy and reliability of the study results, the risk of bias was evaluated in the randomization process and the setting of control reference. Figures 2 and 3 present the proportion of the risk of bias included in the study. The randomization process reflected the low risk of bias, the blind method reflected the unknown and high risk of bias, and the allocation concealment, result evaluation, and other biases showed the unknown risk of bias.

#### Meta‐analysis results of exercise intervention for adolescent depression

##### Meta‐analysis of exercise intervention and adolescent depression

**FIGURE 4:**
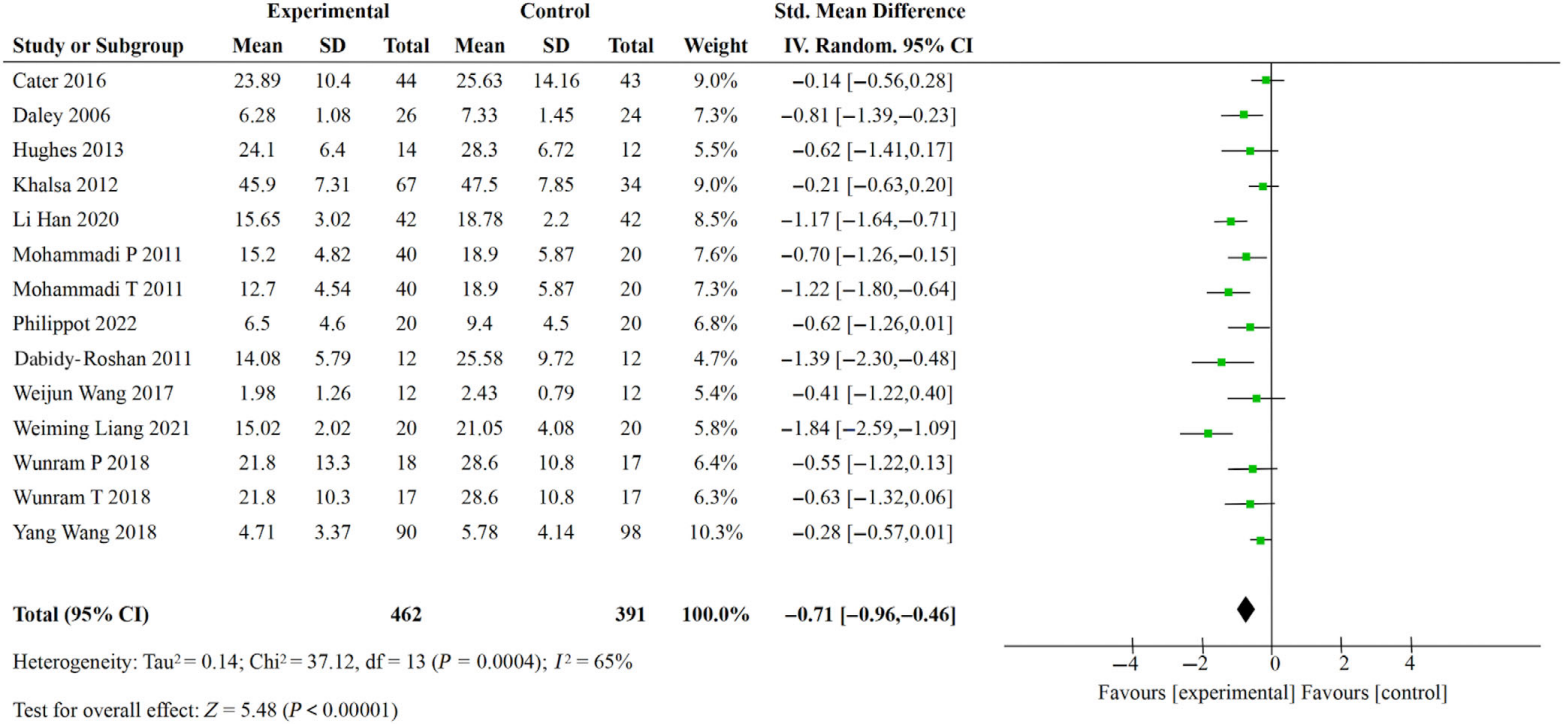
Schematic diagram of meta‐analysis forest of experimental group versus control group of adolescent depression with exercise intervention.

In order to reduce the burden of diseases such as depression and anxiety (Global Burden of Disease 2019 Pakistan Collaborators, 2023), lifestyle changes such as participation in physical activity have been found to be beneficial to health promotion (Pouresmaeil et al., 2019). In this study, the degree of improvement of exercise on depression was analyzed according to the included literature. The results of exercise intervention on adolescent depression were compared between the experimental group and the control group, as shown in Figure 4. Because of the different measurement tools used in various studies, SMD was adopted as the effect size for analysis. The heterogeneity test results showed that *df* = 13 (*p* = .0004) and *I* ^2^ = 65%, indicating there was heterogeneity, so the random effect model was selected for combined analysis. The results illustrated that the effect of exercise intervention on adolescent depression symptoms in the experimental group was significantly better than that in the control group (SMD = −0.71, 95% CI [−0.96, −0.46]). The results indicated that exercise intervention could significantly reduce the 0.71 standard deviation of adolescent depression symptoms.

##### Analysis of publication bias

**FIGURE 5:**
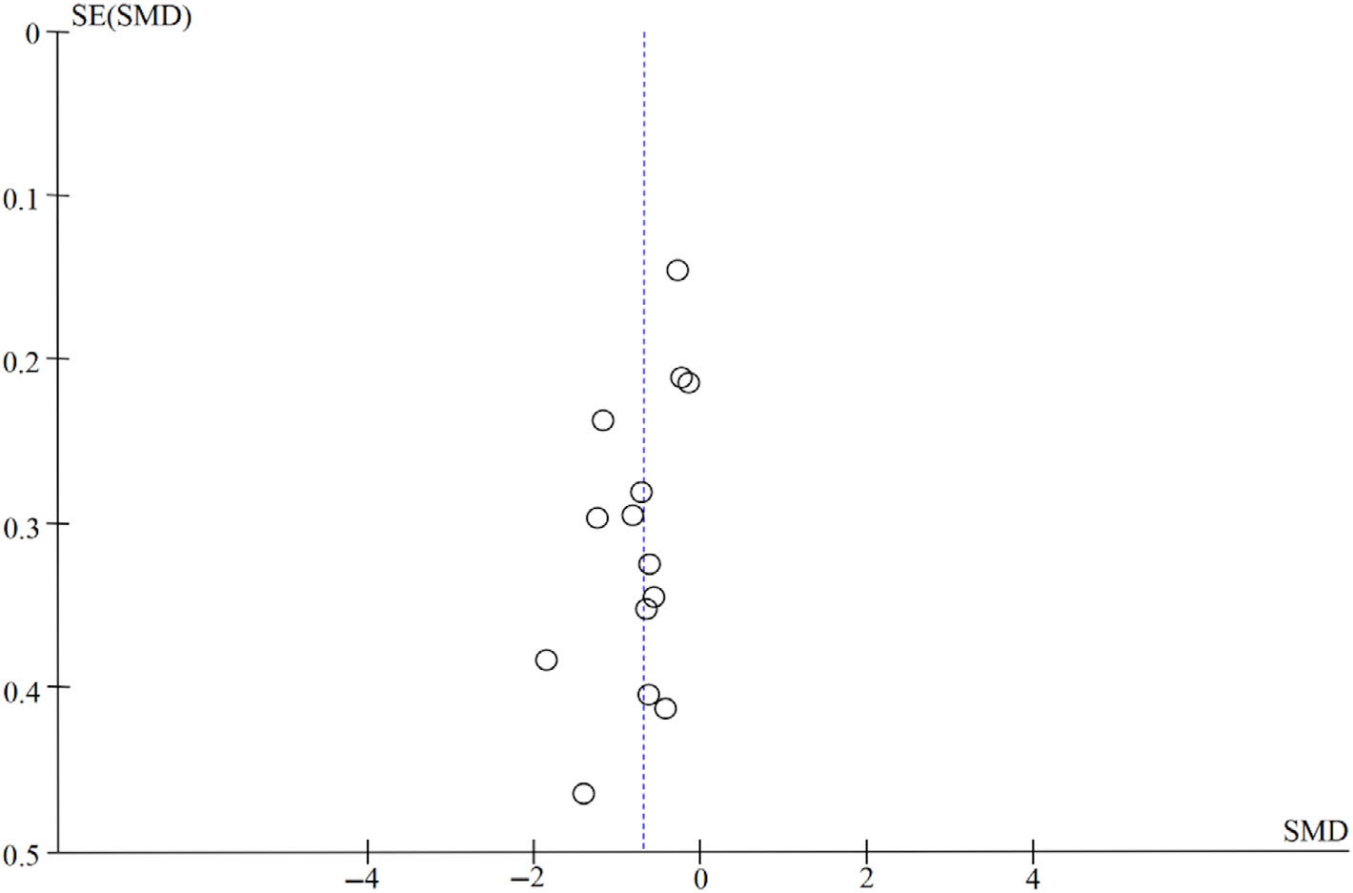
Funnel diagram of the effect of exercise intervention on adolescent depression symptoms. SMD, standardized mean difference. SE, standard error.

A funnel diagram was made of the effect of the level of exercise on improving adolescent depression (as shown in Figure 5), and the results showed that the 12 studies were basically distributed within the confidence interval. The results indicated that publication bias was not obvious, and a meta‐analysis could be conducted. However, one study (Liang et al., 2021) was distributed outside the 95% confidence interval, suggesting that the asymmetry of the funnel diagram may be caused by the heterogeneity among the studies, and further subgroup analysis was needed.

#### Subgroup analysis

In order to explore the influence of regulatory variables on intervention effect, based on the previous studies (Gong et al., 2020), exercise intensity, exercise frequency, single exercise time, total exercise intervention cycle, and exercise type were selected as regulatory variables for subgroup analysis. Among them, exercise intensity was divided into low intensity (heart rate <65% HR_max_), medium intensity (65% HR_max_ < heart rate <75% HR_max_), and high intensity (75% HR_max_ < heart rate <80% HR_max_). The exercise frequency was divided into 2, 3, and 4–5 times per week; the single exercise time was divided into 30–40 min, 45–50 min, and 60–75 min. The total exercise cycle was divided into 4–6 weeks, 8–10 weeks, and 11–14 weeks. The exercise types were divided into group sports and individual sports.

##### Subgroup analysis results

In order to compare whether there was a statistical difference in the pooled effect size of each subgroup in the different studies included, this study quantitatively judged five subgroups of exercise intensity, exercise frequency, exercise time, exercise cycle, and exercise type. According to the analysis of Table 2, exercise intervention depression was influenced by exercise frequency and exercise cycle. At the level of five regulatory variables, except for exercise frequency twice a week, exercise could significantly improve adolescent depression. The details were as follows:

1. Exercise intensity: The effect of low‐intensity exercise on adolescent depression was significant (SMD = −0.68, 95% CI [−1.15, −0.21], *p* = .005), and the effect of medium‐intensity exercise was more significant (SMD = −0.76, 95% CI [−1.30, −0.21], *p* = .006). There was a maximum effect value (SMD = −0.76), but there was no significant difference in the effect value between the tested groups (*p* = .94).
2. Exercise frequency: Two times/week (SMD = −0.14, 95% CI [−0.56, 0.28], *p* = .62) had no significant effect on depression, which may be related to only one study in this subgroup. Both 3 times/week (SMD = −0.84, 95% CI [−1.17, −0.51], *p* = .00001) and 4–5 times/week (SMD = −0.63, 95% CI [−1.10, −0.15], *p* = .010) could significantly improve adolescent depression symptoms, and the effect size (SMD = −0.84) reached the maximum value at the frequency of 3 times/week, and the difference of effect value between the three groups had statistical significance (*p* = .04), namely, 3 times/week had better effect.
3. Exercise time: The exercise intervention lasting for 30–40 min had a significant effect on improving adolescent depression (SMD = −0.47, 95% CI [−0.73, −0.20], *p* = .0005), and the exercise intervention lasting for 45–50 min (SMD = −0.93, 95% CI [−1.59, −0.28], *p* = .005) and 60–75 min (SMD = −0.65, 95% CI [−1.14, −0.15], *p* = .01) had a significant effect on improving adolescent depression, although the maximum effect value (SMD = −0.93) appeared at the duration of 45–50 min. However, there was no significant difference in the effect value between the two groups (*p* = .39).
4. Intervention duration: Exercise intervention with a total period of 4–6 weeks had a significant effect (SMD = −0.80, 95% CI [−1.31, −0.30], *p* = .002), exercise intervention with 8–10 weeks had a significant effect (SMD = −0.95, 95% CI [−1.29, −0.61], *p* < .00001), and exercise intervention with 11–14 weeks had a significant effect (SMD = −0.37, 95% CI [−0.62, −0.13], *p* = .002). The maximum effect size of exercise for 8–10 weeks (SMD = −0.95) significantly differed among the groups (*p* = .02). Namely, exercise intervention for 8–10 weeks had the best effect.
5. Exercise type: Group exercise intervention had a significant effect (SMD = −0.75, 95% CI [−1.07, −0.44], *p* < .00001), and individual exercise (SMD = −0.60, 95% CI [−1.06, −0.13], *p* = .01) also had a significant effect on improving adolescent depression. Among them, aerobic exercise possessed the maximum effect value (SMD = −0.75), but the difference between the two groups was not significant (*p* = .58).

**TABLE 2:** Subgroup analysis results of exercise intervention group versus control group.

Moderators	Level	Number of literature (*N*)	SMD	CI	*I* ^2^	Heterogeneity test between groups		
						*Q*	*df*	*p*
Exercise intensity	Low	2	−0.68**	−1.15, −0.21	0	0.12	2	.94
	Medium	5	−0.76**	−1.30, −0.21	81			
	High	2	−0.63*	−1.15, −0.10	0			
Exercise frequency	2 times/week	1	−0.14	−0.56, 0.28	NA	6.59	2	.04
	3 times/week	7	−0.84**	−1.17, −0.51	59			
	4–5 times/week	4	−0.63*	−1.10, −0.15	71			
Exercise time	30–40 min	4	−0.47**	−0.73, −0.20	0	1.86	2	.39
	45–50 min	4	−0.93**	−1.59, −0.28	86			
	60–75 min	3	−0.65**	−1.14, −0.15	67			
Intervention duration	4–6 weeks	5	−0.80**	−1.31, −0.30	72	7.95	2	.02
	8–10 weeks	3	−0.95**	−1.29, −0.61	28			
	11–14 weeks	4	−0.37**	−0.62, −0.13	16			
Exercise Type	Team sports	10	−0.75**	−1.07, −0.44	69	0.31	1	.58
	Individual sports	4	−0.60**	−1.06, −0.13	57			

Abbreviations: CI, 95% confidence interval; SMD, standardized mean difference.

* *p* < .05;

** *p* < .01.

#### Sensitivity analysis

Sensitivity analysis was carried out by changing the research model and eliminating the studies one by one. After converting the random effect model to the fixed effect model, it was found that the combined effect of the random effect model was (SMD = −0.71, 95% CI [−0.96, −0.46]), and the combined effect of the fixed effect model was (SMD = −0.59, 95% CI [−0.73, −0.45]), suggesting that the difference between the two was relatively small. After eliminating the included studies one by one, it was found that the combined effect size was between −0.63 and −0.76 (see Table 3 for the specific process). The above two methods indicated that the analysis of this study had good stability.

**TABLE 3:** Combined effect and confidence interval after eliminating the included literature.

Excluded document	SMD	95% CI	*p*	*I* ^2^
Carter 2016	−0.76	−1.03, −0.50	<.0001	63%
Daley 2006	−0.70	−0.97, −0.43	<.0001	67%
Hughes 2013	−0.72	−0.98, −0.45	<.0001	68%
Khalsa 2012	−0.76	−1.03, −0.49	<.0001	64%
Mohammadi P 2011	−0.71	−0.99, −0.44	<.0001	68%
Mohammadi T 2011	−0.67	−0.92, −0.41	<.0001	63%
Philippot 2022	−0.72	−0.99, −0.45	<.0001	68%
Dabidy‐Roshan 2011	−0.67	−0.93, −0.42	<.0001	65%
Wunram P 2018	−0.72	−0.99, −0.45	< .0001	68%
Wunram T 2018	−0.72	−0.99, −0.45	<.0001	68%
Liang 2021	−0.63	−0.85, −0.40	<.0001	54%
Wang 2017	−0.73	−0.99, −0.46	<.0001	68%
Wang & Liu 2018	−0.76	−1.03, −0.49	<.0001	62%
Han 2020	−0.66	−0.91, −0.41	<.0001	61%

Abbreviations: CI, 95% confidence interval.

### Discussion

After analyzing 12 articles, the combined effect of this study was (SMD = −0.71, 95% CI [−0.96, −0.46]), which indicated that exercise could significantly improve the depression symptoms of adolescents aged 12–18 years old. Compared with the research results (SMD = −1.26, 95% CI [−1.69, −0.82]) of Wang and Wan (2019), the reason might be that the research objects were 18–24 year‐old college students, had a high level of physical activity, and relatively good physical quality. Therefore, the effect size was higher than that of this study.

1. Exercise intensity: Since there was a certain heterogeneity (*I* ^2^ = 65%) among the literature in this study, the random effect model was used for analysis. Afterward, the regulatory variables were determined through subgroup analysis to explore the causes of heterogeneity. Exercise intensity was one of the key factors in formulating exercise prescriptions for depressed adolescents. In this study, the effect of exercise intensity on improving depression symptoms of adolescents was analyzed. It was found that low‐intensity, medium‐intensity, and high‐intensity exercise had significant improvement effects, but the effect size of medium‐intensity exercise was larger than that of low‐intensity and high‐intensity exercise. Although it has been found that the depression of adolescents, such as female college students, significantly improved after low‐intensity exercise interventions (Pei, 2018; Wang & Yan, 2006; Zheng, 2015; Zheng, 2020), some studies also confirmed that low‐intensity exercise did not significantly improve the depression of adolescents (male college students, etc.) (Balchin et al., 2016; Mao & Shen, 2014; Paolucci et al., 2018; Saltan & Ankaralı, 2021). The results indicated that the effect of low‐intensity exercise on improving adolescent depression remains to be clarified. Alternatively, the medium intensity was the first choice in the formulation of exercise prescription for depressed adolescents; the comparative study demonstrated that its effect on improving depression symptoms or depression mood of adolescents (male/female college students, high‐school students) was significantly better than that of low intensity and high‐intensity exercise. The reason was that medium‐intensity exercise could significantly promote the secretion of neurotransmitters such as 5‐HT and DA and hormones such as GC, CRH, and ACTH, as well as inhibit IL‐6 and TNF‐α mediate inflammatory reactions (Chen et al., 2022). To sum up, medium intensity was the first choice of exercise intensity when employing exercise to improve adolescent depression.
2. Exercise frequency: The exercise frequency was divided into 2 times/week, 3 times/week, and 4–5 times/week according to the included situations. It was found that the difference between the 2 times/week group was not significant. At present, the research on exercise frequency is still controversial because some studies confirmed that 2 times/week softball, Sanda training, Tai Chi, badminton, and dance could obviously improve the depression of college students and vocational nursing students (Guo, 2020; Pan, 2019; Qin et al., 2018). However, Zheng (2015) found that the results of badminton twice a week on female college students' depression were not effective, which was closely related to the low intensity of exercise. Compared with medium‐intensity and high‐intensity exercise, low‐intensity exercise had no significant effect on improving adolescent depression (Balchin et al., 2016). In this study, 3 times/week and 4–5 times/week exhibited significant inter‐group differences in improving adolescent depression, and 3 times/week had the largest effect size. The result was consistent with the best effect of 3 times/week physical exercise on depression found by Wang et al. (2018). After exercising at the frequency of 3 times/week, the depression level or depression mood of male and female college students significantly improved (Balchin et al., 2016; Mao & Shen, 2014). Although the exercise frequency of 4–5 times/week also had a significant effect on improving depression in adolescents (Wu, 2019), due to the COVID‐19 pandemic, Internet addiction, and lifestyle changes, adolescents have less free time due to high academic pressure (Global Burden of Disease 2021 Health Financing Collaborator Network, 2023; Lebni et al., 2020). In view of the reasons such as high academic pressure of adolescents leading to less free time, high exercise frequency leading to sports injury, and the maximum effect amount of exercise frequency of 3 times/week, the exercise frequency should be 3 times/week when formulating the exercise prescription for adolescent depression.
3. Exercise time: Exercise time was directly related to the effectiveness of exercise prescription for depressed adolescents. It has been confirmed that exercise interventions of 30, 40, 45, 50, 60, 80, and 90 min/time could obviously improve the depression symptoms of adolescents (male/female college students, junior high‐school students) (Chen et al., 2020; Fan, 2020; Guo, 2020; Pei, 2018; Wang, 2021; Wang & Xie, 2016; Zheng, 2020). In this study, according to the characteristics of the included literature, the exercise time was divided into three groups: 30–40 min, 45–50 min, and 60–75 min (Gong et al., 2020). It was found that there were significant differences among the three groups, but the single intervention time of 45–50 min had the largest effect size, followed by 60–75 min, and 30–40 min had the smallest effect size. Relatively shorter exercise time would reduce the amount of exercise for depressed adolescents, while longer exercise time would lead to excessive exercise. When the amount of exercise was small or large, the improvement effect on adolescent depression would be reduced (Wang et al., 2022). Moreover, youth groups were mainly junior high‐school students, senior high‐school students and college students, and the related sports intervention experiments were often in physical education classes. Therefore, the exercise time of the intervention for adolescent depression should be 45–50 min/time.
4. Program duration: According to the characteristics of the included literature, the total exercise cycle was divided into 4–6 weeks, 8–10 weeks, and 11–14 weeks. When factors such as exercise intensity and exercise type were not considered, exercise duration of 4, 6, 8, 12, 14, and 16 weeks could significantly improve the depressive symptoms of adolescents, and the frequency of exercise at 12 weeks was the highest (Paolucci et al., 2018; Saltan & Ankaralı, 2021; Wang & Xie, 2016; Wang & Yan, 2006; Zhao, 2017). By analyzing the included literature, it could be found that the exercise cycle of 4–6 weeks, 8–10 weeks, and 11–14 weeks had significant inter‐group differences, indicating that the exercise cycle of 4–14 weeks could significantly improve adolescent depression symptoms, which was consistent with the previous studies. In addition, it was found that 8–10 weeks had the maximum effect size. Reinodt et al. (2022), You et al. (2021), and Wunram et al. (2018) confirmed that exercise intervention (running, cycling, and calisthenics) for 8, 9, and 10 weeks could significantly improve the depression symptoms of adolescents. The results indicated that this exercise cycle had the best performance in improving adolescent depression.
5. Exercise type: In the meta‐analysis of exercise intervention, the classification of exercise type was affected by the included literature and the group characteristics of the subjects. A total of 12 articles were included in this study. The specific types of exercises included aerobic exercise, yoga, aerobics, football, volleyball, and so forth; these exercises focused mainly on aerobic exercise and these exercises included strength training and sports games. However, there were obvious differences between the different types of exercises, and it was not easy to reflect the characteristics of each type of sports in the adjustment variable group. Moreover, the subjects in this study were adolescents with different degrees of depression, often accompanied by many non‐communicable diseases, solitary personality, poor interpersonal communication, low self‐esteem, and social anxiety (Global Burden of Disease 2019 Iran Collaborators, 2022; Schmidt et al., 2022). In contrast, participating in sports activities could help improve the interpersonal communication ability of adolescents and improve depression (Sun & Zhang, 2020). Therefore, in combination with the above two aspects, the presented study divided the types of sports into group sports and individual sports. Compared with individual sports, group sports could better promote depressed adolescents to participate in social activities, cultivate the ability of unity and cooperation, as well as the spirit of collectivism, and therefore better reduce the negative psychological emotions caused by depression (Mohammadi & Abhar, 2011). In this study, group exercise was adopted in ten studies. From the results of subgroup analysis (SMD = −0.75, 95% CI [−1.07, −0.44], *p* < .00001), the effect of group exercise intervention was better than that of individual exercise (SMD = −0.60, 95% CI [−1.06, −0.13], *p* = .01). It was suggested that both group exercise and individual exercise could prominently improve the depression symptoms of adolescents, but group exercise exhibited better performance.

During the meta‐analysis of this study, some studies were excluded due to missing data and the inability to download the full text, so only 12 articles were finally included. In the process of subgroup analysis, the grouping criteria of moderator variables were affected by some studies, and some groups included only one study, which may bias the results. At the same time, the literature included in this study used different scales for the diagnostic measurement of depression, resulting in the inability to achieve uniform outcome indicators, which may have a certain impact on the final results.

## CONCLUSION

Through the analysis of research in China and elsewhere, this study found that the effect of exercise on improving adolescent depression is controversial. Based on this, this study put forward the concept of “precision exercise” and used meta‐analysis to analyze the current research on the effect of exercise on adolescent depression, and found that: (1) the intensity of exercise should be moderate; (2) exercise frequency of 3 times per week is the most effective in improving adolescent depression, and the interval of each intervention should be set reasonably to ensure rest; (3) exercise time should be 45–50 min/time; (4) exercise cycles of 8–10 weeks of exercise can improve adolescent depressive symptoms; (5) collective exercise, such as aerobics, has the best effect. The movement time and period can vary depending on the actual situation. In conclusion, moderate‐intensity aerobic exercise (45–50 min/session, 3 sessions/week, for 8–10 weeks) can significantly improve adolescent depressive symptoms.

Currently, it is necessary to strengthen the cross‐sectional research on the causes and prevalence of adolescent depression, especially in college and high‐school students. In addition, empirical studies on exercise intervention for different types of adolescent depression and age groups are needed to detect and verify the effects of different exercise intensity, exercise frequency, exercise time, exercise type, and exercise cycle on adolescent depression and to establish and gradually improve the precision exercise prescription database for adolescent depression. In addition, virtual simulation technology can also be used to realize the visualization and popularization of the types, causes, and prevention and treatment of exercise of adolescent depression so as to provide easy‐to‐understand materials for universities, primary and secondary schools, hospitals, and other organizations to strengthen the publicity of exercise prevention and treatment of adolescent depression.

## CONFLICT OF INTEREST STATEMENT

The authors declare there are no conflicts of interest.

## Data Availability

All data used in this study are cited in the manuscript.
